# Datasets on chemical composition and anaerobic digestion of organic fraction of municipal solid waste (OFMSW), digested sewage sludge (inoculum) and ashes from incineration or gasification

**DOI:** 10.1016/j.dib.2020.105797

**Published:** 2020-06-02

**Authors:** Gregor Sailer, Johanna Eichermüller, Jens Poetsch, Sebastian Paczkowski, Stefan Pelz, Hans Oechsner, Joachim Müller

**Affiliations:** a University of Applied Forest Sciences Rottenburg, Schadenweilerhof, 72108 Rottenburg, Germany; b State Institute of Agricultural Engineering and Bioenergy, University of Hohenheim, Garbenstrasse 9, 70599 Stuttgart, Germany; c Institute of Agricultural Engineering, Tropics and Subtropics Group, University of Hohenheim, Garbenstrasse 9, 70599 Stuttgart, Germany

**Keywords:** OFMSW, Sewage sludge, Wood ashes, Waste characterization, Energy potentials, Trace elements

## Abstract

This article contains data on the chemical composition and anaerobic digestion of different residue streams including OFMSW, digested sewage sludge, low-carbon (LC) ashes from incineration subdivided into LC coarse and LC fly ash as well as high-carbon (HC) ashes from gasification subdivided into HC reactor and HC fly ash. All materials were collected in accordance to standard procedures in southern Germany. The data presented in this article include (1) dry matter (2) organic dry matter (3) elemental analysis (4) trace elements and (5) cumulative biogas and CH_4_ yields. Researchers and waste management companies on lab-/pilot-/industrial-scale can rely on the presented data for classification and comparison of biogenic waste streams. For further discussion, please refer to the scientific article entitled **“Optimizing anaerobic digestion of organic fraction of municipal solid waste (OFMSW) by using biomass ashes as additives” [****1****]**.

**Specifications table**

**Table:** 

**Subject**	Environmental Engineering
**Specific subject area**	Waste characterization, optimizing anaerobic digestion and potentials of biomass ashes, creation of synergies by combined utilization of residue streams
**Type of data**	15 tables
**How data were acquired**	Datasets for OFMSW, digested sewage sludge, low-carbon (LC) and high-carbon (HC) ashes were acquired using typical physico-chemical analyzes and instruments: Fresh mass (FM) and dry matter (DM) through oven drying (UNP 700, Memmert, Schwabach, Germany) Organic dry matter (oDM) through muffle furnace (AAF 1100, Carbolite, Neuhausen, Germany) C, H, N through elemental analysis (vario MACRO cube, elementar, Langenselbold, Germany) Trace elements (TE) through inductively coupled plasma-optical emission spectroscopy (ICP-OES) (Spectro Blue, ASX-260 auto sampler, SPECTRO Analytical Instruments, Kleve, Germany) Biogas and CH_4_ yields through batch-digesters (own construction, [1]) CH_4_ concentrations were measured by a portable biogas monitor (BIOGAS 5000, Geotech, Coventry, UK)
**Data format**	Raw, processed (mean values)
**Parameters for data collection**	After collection in 2018/2019 and processing (drying, sorting, sieving, crushing), each sample was stored airtight as DM until further experiments were carried out (from February to June 2019). All analyzes were performed with 3–6 repetitions. All digestion experiments were conducted at 35 °C considering temperatures within the digesters and ambient conditions. Biogas and CH_4_ yield data were expressed on standard conditions (1013 hPa, 0 °C, dry gas).
**Description of data collection**	Extensive waste characterization with the aim to create synergy effects in the treatment (combined treatment) of residue streams origin from different processes. The following data, inter alia, include: FM, DM, oDM and in total 37 elements (C, H, N, O, Al, Ag, As, B, Ba, Be, Bi, Ca, Cd, Co, Cr, Cu, Fe, K, Li, Mg, Mn, Mo, Na, Ni, Pb, Sb, Se, Sr, Ti, Tl, V, Zn, Ga, In, Si, P, S). Through batch tests in triplicate (series 1) and duplicate (series 2), the anaerobic digestion behavior and the impact of wood ashes in nine different configurations (mixing ratios) was measured.
**Data source location**	All materials were collected in the state of Baden-Württemberg (southern Germany)
**Data accessibility**	Data are available in this article
**Related research article**	Sailer, G.; Eichermüller, J.; Poetsch, J.; Paczkowski, S.; Pelz, S.; Oechsner, H.; Müller, J., 2020, Optimizing anaerobic digestion of organic fraction of municipal solid waste (OFMSW) by using biomass ashes as additives, Waste Management 109 (2020), 136–148, doi: 10.1016/j.wasman.2020.04.047.

**Value of the data**

• This data article provides a large characterization of six highly relevant waste types (OFMSW, digested sewage sludge as well as wood ashes from incineration and gasification) and demonstrates the impact of wood ashes on the anaerobic digestion of OFMSW.
• This data will be useful for other researchers in the field of bio- and thermo-chemical biomass conversion (comparison, data basis). Managers of biogenic waste streams, biogas plant operators can rely on the data for optimization and identification of waste treatment concepts.
• Researchers and developers in the fields of treatment, recycling and disposal of bio-waste (lab-, pilot-, and industrial-scale) can rely on the presented data for classification and comparison of data and replication/variation of the conducted experiments.
• In general, data on the characteristics and bio-chemical treatment of German OFMSW and ashes from wood gasification processes are lacking. Datasets are relevant for the determination of practical application and synergy possibilities of OFMSW, sewage sludge and biomass ashes.

## 1 Data description

This Data in Brief article provides the raw data for chemical composition and anaerobic digestion of OFMSW together with digested sewage sludge and low-carbon (LC) ashes from incineration as well as high-carbon (HC) ashes from gasification. All data are presented within this article.

Table 1 defines acronyms and additional information for all materials. Tables 2 and 3 present all raw data and weighted mean values for fresh mass (FM) [g], dry matter (DM) [% FM] and organic dry matter (oDM) [% DM] contents. Table 4 shows measured raw data and mean values for C, H and N [% DM]. Based on C, H and N, stoichiometric biogas and CH_4_ yields can be calculated according to [2, 3]. Values for S can be obtained by using the data from the inductively coupled plasma-optical emission spectroscopy (ICP-OES, Table 8). O [% DM] is defined as 100% subtracted by the contents [% DM] of C, H, N, S and ash. By including oDM data of Table 3, O can be calculated. Single and mean values for 33 trace elements (TE) (Al, Ag, As, B, Ba, Be, Bi, Ca, Cd, Co, Cr, Cu, Fe, K, Li, Mg, Mn, Mo, Na, Ni, Pb, Sb, Se, Sr, Ti, Tl, V, Zn, Ga, In, Si, P, S) measured by ICP-OES are presented in Table 5-8.

**Table 1:** Acronyms for each material and annotations.

**Material**	**Annotation**	**Acronym**
OFMSW	Separately collected; coarse impurities (stones, metals, plastics) were manually removed before further processing and analytics	OFMSW
Sewage sludge (digested)	Used as inoculum for digestion experiments. Treatment of wastewater and storage of sewage sludge at a mesophilic temperature of 37 °C at the sewage plant	SEWS
HC fly ash	From a full-scale wood gasification plant (fixed bed, 150 kW_el_, 300 kW_th_)	HC-FA
HC reactor ash	From a full-scale wood gasification plant (fixed-bed, 150 kW_el_, 300 kW_th_)	HC-RA
LC fly ash	From a full-scale heating plant (50 MW, grate firing) using landscape management material and forest residues (50:50)	LC-FA
LC coarse ash	From a full-scale heating plant (50 MW, grate firing) using landscape management material and forest residues (50:50)	LC—CA

**Table 2:** Raw data and mean values for DM contents and total amount of FM in each material.

**Material**	**Annotation**	**FM** **[g]**	**DM** **[% FM]**
OFMSW	Mainly food waste	1450	21.93
OFMSW	Mixture	1397	37.01
OFMSW	Mixture	2833	34.27
OFMSW	Mixture	1968	34.15
OFMSW	Mixture	2897	35.59
**Weighted mean**		**10,545**	**33.28**
SEWS	Digestion experiments, test series 1	43.13	3.96
SEWS	Digestion experiments, test series 1	49.30	3.99
SEWS	Digestion experiments, test series 1	47.03	3.98
**Weighted mean**		**139.46**	**3.98**
SEWS	Digestion experiments, test series 2	20.98	3.26
SEWS	Digestion experiments, test series 2	20.68	3.26
SEWS	Digestion experiments, test series 2	27.12	3.26
**Weighted mean**		**68.78**	**3.26**
HC-FA		311.33	22.59
HC-RA		285.47	24.46
LC-FA	Samples were provided as DM	–	–
LC—CA	Samples were provided as DM	–	–

**Table 3:** Raw data and mean values for oDM contents in each material.

**Material**	**Annotation**	**oDM** **[% DM]**
OFMSW		75.89
OFMSW		76.83
OFMSW		78.70
OFMSW		79.77
OFMSW		78.21
**Mean**		**77.88**
SEWS	Digestion experiments, test series 1	64.23
SEWS	Digestion experiments, test series 1	64.02
SEWS	Digestion experiments, test series 1	64.03
**Mean**		**64.09**
SEWS	Digestion experiments, test series 2	63.13
SEWS	Digestion experiments, test series 2	63.00
SEWS	Digestion experiments, test series 2	63.03
**Mean**		**63.07**
HC-FA		73.60
HC-FA		73.30
HC-FA		73.65
**Mean**		**73.51**
HC-RA		75.85
HC-RA		77.50
HC-RA	Measured value (52.72) defined as outlier and discarded	–
**Mean**		**76.68**
LC-FA		0.03
LC-FA		0.05
LC-FA	Measured value (−0.07) not plausible and discarded	–
**Mean**		**0.04**
LC—CA		0.91
LC—CA		0.89
LC—CA		0.71
**Mean**		**0.84**

**Table 4:** Raw data and mean values for C, H and N in all materials. For determination of stoichiometric CH_4_ yields, S can be calculated by using data from ICP-OES (Table 8). O is defined as 100% DM - X_C, H, N, S, Ash [% DM]_. SEWS represented a composite sample of SEWS (test series 1) and SEWS (test series 2).

**Material**	**C** **[% DM]**	**H** **[% DM]**	**N** **[% DM]**
OFMSW	42.17	5.64	2.21
OFMSW	40.58	5.46	2.59
OFMSW	35.32	4.72	1.71
OFMSW	39.90	5.34	2.00
**Mean**	**39.49**	**5.29**	**2.13**
SEWS	29.83	4.36	4.02
SEWS	29.60	4.40	3.37
SEWS	29.84	4.43	3.74
SEWS	29.45	4.40	3.81
**Mean**	**29.68**	**4.40**	**3.83**
HC-FA	68.90	0.75	1.16
HC-FA	70.68	0.77	0.74
HC-FA	70.12	0.77	0.32
HC-FA	68.78	0.75	0.51
**Mean**	**69.62**	**0.76**	**0.68**
HC-RA	73.71	0.72	0.62
HC-RA	73.47	0.76	0.65
HC-RA	73.56	0.77	0.55
HC-RA	73.29	0.76	0.60
**Mean**	**73.51**	**0.75**	**0.61**
LC-FA	2.76	0.23	0.14
LC-FA	2.22	0.24	0.16
LC-FA	1.87	0.22	0.15
LC-FA	2.54	0.23	0.10
**Mean**	**2.35**	**0.23**	**0.14**
LC—CA	1.32	0.29	0.11
LC—CA	1.26	0.40	0.18
LC—CA	1.13	3.23 (discarded)	0.18
LC—CA	1.15	0.23	0.74 (discarded)
**Mean**	**1.22**	**0.31**	**0.16**

**Table 5:** Raw data and mean values for TE measured by ICP-OES. Values marked with * were at detection limit. All values relate to mg/kg DM.

**Material**	**Al**	**Ag**	**As**	**B**	**Ba**	**Be**	**Bi**	**Ca**	**Cd**
OFMSW	6084.76	0.21	2.39	2.12*	88.40	0.02*	0.66	28,270.76	discarded
OFMSW	7345.80	0.02*	2.32	2.13*	86.59	0.02*	1.20	43,638.44	0.41
OFMSW	6247.57	0.14	4.79	2.17*	88.11	0.02*	0.02*	25,320.14	0.20
OFMSW	5546.85	0.32	1.86	2.00*	74.20	0.02*	0.02*	21,512.88	0.15
**Mean**	**6306.25**	**0.17**	**2.84**	**2.11**	**84.32**	**0.02**	**0.47**	**29,685.55**	**0.25**
SEWS	19,789.67	1.01	4.17	2.14*	627.72	0.02*	3.54	47,342.26	1.45
SEWS	20,125.29	0.98	4.17	2.19*	654.66	0.02*	3.66	49,099.45	1.49
SEWS	19,310.18	1.22	4.20	2.21*	621.15	0.02*	2.90	47,217.84	1.38
SEWS	18,884.97	1.10	4.18	2.16*	612.33	0.02*	3.20	46,743.74	1.33
**Mean**	**19,527.53**	**1.08**	**4.18**	**2.18**	**628.97**	**0.02**	**3.32**	**47,600.82**	**1.41**
HC-FA	697.63	1.66	0.06*	6.63*	633.73	0.06*	0.06*	103,964.50	2.90
HC-FA	649.94	2.39	0.06*	6.85*	624.24	0.06*	0.06*	101,162.79	2.63
HC-FA	648.65	2.96	0.06*	7.21*	597.88	0.06*	0.06*	98,455.60	2.32
HC-FA	499.20	1.70	0.05*	5.59*	482.97	0.05*	0.05*	76,773.23	1.80
**Mean**	**623.86**	**2.18**	**0.06**	**6.57**	**584.70**	**0.06**	**0.06**	**95,089.03**	**2.41**
HC-RA	434.32	1.36	0.04*	5.00*	289.90	0.04*	0.04*	60,277.03	0.04*
HC-RA	1151.61	2.17	0.12*	13.90*	771.71	0.12*	0.12*	159,925.56	0.12*
HC-RA	567.03	0.14	0.06*	6.47*	378.00	0.06*	0.06*	76,501.15	0.06*
HC-RA	476.91	0.05*	0.05*	5.93*	346.72	0.05*	0.05*	103,125.00	0.05*
**Mean**	**657.47**	**0.93**	**0.07**	**7.82**	**446.58**	**0.07**	**0.07**	**99,957.19**	**0.07**
LC-FA	24,309.57	0.03*	25.05	269.97	1617.49	0.03*	0.03*	237,062.71	8.48
LC-FA	25,144.84	0.03*	25.10	272.78	1500.66	0.03*	0.03*	231,580.69	8.86
LC-FA	24,971.34	0.03*	23.65	260.11	1459.82	0.03*	0.03*	218,610.01	8.14
LC-FA	25,322.28	0.03*	21.32	276.43	1917.11	0.03*	0.03*	186,472.15	7.76
LC-FA	25,943.27	0.03*	22.33	278.56	1936.68	0.03*	0.03*	194,854.88	8.64
LC-FA	26,290.62	0.03*	20.77	266.02	1918.43	0.03*	0.03*	186,690.89	7.79
**Mean**	**25,330.32**	**0.03**	**23.04**	**270.64**	**1725.03**	**0.03**	**0.03**	**209,211.89**	**8.28**
LC—CA	24,186.22	0.03*	6.06	93.90	732.70	0.03*	0.03*	140,936.06	0.10
LC—CA	24,115.81	0.03*	6.30	85.70	695.54	0.03*	0.03*	143,766.40	0.10
LC—CA	23,873.10	0.03*	6.69	83.32	711.93	0.03*	0.03*	145,748.19	0.10
LC—CA	24,370.87	0.03*	6.44	72.32	866.25	0.03*	0.03*	171,796.57	0.07
LC—CA	24,219.55	0.03*	6.95	75.44	843.98	0.03*	0.03*	162,442.40	0.10
LC—CA	24,871.74	0.03*	discarded	75.88	784.27	0.03*	0.03*	160,509.14	0.07
**Mean**	**24,272.88**	**0.03**	**6.49**	**81.10**	**772.44**	**0.03**	**0.03**	**154,199.79**	**0.09**

Table 9–11 present data for the first digestion experiment subdivided into the experimental set-up (Table 9), measured CH_4_ concentrations (Table 10) and cumulative gas yields for each time step and digester (Table 11). Table 12–15 show data for the second digestion experiment subdivided into the experimental set-up (Table 12), measured CH_4_ concentrations (Table 13) and cumulative gas yields for each time step and digester (Tables 14 and 15).

## 2 Experimental design, materials and methods

Detailed descriptions for all experiments can be found in the original research paper [1].

### 2.1 Sampling

OFMSW (untreated) was collected at a full-scale thermophilic plug-flow biowaste fermentation plant in southern Germany according to standard procedures defined in the German biowaste ordinance [4] Digested sewage sludge as residue after anaerobic digestion of wastewater (used as inoculum for digestion experiments) was collected at the local municipal sewage treatment plant (Rottenburg-Kiebingen, Germany) following [5]. LC coarse and LC fly ash from a 50 MW heating plant in southern Germany with a grate fired furnace using a fuel mixture of 50:50 landscape management material and forest residues was provided within another project [6]. HC reactor (minor quantities) and fly ash (major proportion) from a full-scale fixed-bed wood gasifier (Konstanz-Mainau, Germany) fueled with untreated natural wood chips were provided by the plant operator (sampling according to standard procedures). Only HC fly ash was used for digestion experiments because this fraction showed a higher relevance from a disposal perspective. However, both ashes were characterized in order to gain insights in special wood ashes and to compare their TE profiles with OFMSW and sewage sludge. An overview of all materials is provided in Table 1.

### 2.2 DM, processing and oDM

DM (Table 2) was determined through drying at 105 °C in a drying oven for at least 24 h [7]. The fresh OFMSW sample was manually sorted into fractions to determine the DM content for food waste separately. Before further processing, impurities were removed and the remaining DM was re-combined as well as milled to particle sizes of approximately 1 mm with a cutting mill. The dry sewage sludge was manually crushed in a ceramic mortar (no prior sorting was required). From the dry LC coarse ash, impurities (metals) were removed by manual sorting and sieving with mesh sizes of 16 mm, 8 mm and 3.15 mm. The remaining DM was ground to a particle size of 1 mm by a jaw crusher followed by an orbital mono mill. The dry HC reactor ash was milled by an orbital mono mill to particle size of 1 mm without prior sorting. The particle sizes of HC and LC fly ashes already were below 1 m. Therefore, no further treatment was necessary. oDM (Table 3) was determined by using approximately 1 g of DM in a ceramic crucible by a muffle furnace [8].

### 2.3 C, H, N, S, O

Elemental analysis (C, H, N) was carried out [9] for all materials containing oDM (sewage sludge, OFMSW and HC ashes; Table 4). Approximately 40 mg per sample were pressed into a zinc foil coated tablet. S was not measured simultaneously in favor of the measurement accuracy of C, H and N (S was measured via ICP-OES). The measured values for C, H, N, S and ash were used to determine O contents.

### 2.4 TE

TE (Tables 5, 6, 7, 8) were measured via ICP-OES [10] after digestion in aqua regia. Therefore, 300 mg DM per sample were transferred into 50 mL Teflon vessels and combined with 1 mL H_2_O_2_. Before microwave digestion at 190 °C, 3 mL HNO_3_ (69%) and 9 mL HCl (35%) were added. The digested residues were aliquoted to 50 mL with aqua bidest and measured at the ICP-OES system. Solid residues (Si) were separated by a centrifuge before the spectroscopy and their weight was deducted from the sample weight. Therefore, values for Si only represented a partial amount of the total amount (not completely digestible in aqua regia). When evaluating ICP-OES data, all values below the detection limit were equated with this limit. Hence, some of those values might be slightly overestimated as the actual values could be even lower than the detection limit (0 < value < detection limit).

**Table 6:** Raw data and mean values for TE measured by ICP-OES. Values marked with * were at detection limit. All values relate to mg/kg DM.

**Material**	**Co**	**Cr**	**Cu**	**Fe**	**K**	**Li**	**Mg**	**Mn**	**Mo**
OFMSW	1.38	10.96	17.71	4446.15	13,646.09	21.74	2890.78	160.68	0.87
OFMSW	1.35	11.24	15.72	5518.99	12,589.73	13.23	3437.71	172.58	0.72
OFMSW	1.47	13.64	16.61	4995.93	13,709.64	28.74	2817.42	189.87	0.81
OFMSW	1.06	11.68	16.97	3808.83	12,325.73	21.61	2557.94	142.22	0.82
**Mean**	**1.32**	**11.88**	**16.75**	**4692.48**	**13,067.80**	**21.33**	**2925.97**	**166.34**	**0.81**
SEWS	4.09	55.12	521.53	67,361.38	10,349.33	28.20	10,206.12	174.70	4.89
SEWS	4.31	56.87	543.19	70,555.99	11,008.61	30.01	10,506.85	180.91	5.25
SEWS	4.14	59.37	503.67	66,258.88	11,439.42	24.98	10,309.59	173.82	5.68
SEWS	3.93	56.36	494.34	65,664.74	10,561.66	20.94	10,088.44	169.36	6.05
**Mean**	**4.12**	**56.93**	**515.68**	**67,460.25**	**10,839.76**	**26.03**	**10,277.75**	**174.70**	**5.47**
HC-FA	0.41	3.67	73.96	1039.05	35,460.15	2.28	15,632.54	3107.10	0.06*
HC-FA	0.12	4.53	61.69	903.30	32,058.54	4.25	14,739.90	3045.90	0.06*
HC-FA	0.19	4.18	58.82	914.41	44,440.57	4.60	14,519.95	3013.51	0.06*
HC-FA	0.05*	4.15	51.45	738.26	29,316.51	2.72	11,158.34	2261.74	0.05*
**Mean**	**0.19**	**4.13**	**61.48**	**898.76**	**35,318.94**	**3.46**	**14,012.68**	**2857.06**	**0.06**
HC-RA	0.04*	13.49	47.52	1458.45	39,071.13	3.15	8271.67	1163.54	0.04*
HC-RA	0.12*	39.83	110.61	3997.52	103,518.86	6.51	22,696.03	3074.44	0.12*
HC-RA	0.06*	17.84	56.50	1887.41	50,337.30	2.86	10,885.68	1493.07	0.06*
HC-RA	0.05*	13.29	46.11	704.45	60,080.08	3.95	10,618.11	1493.64	0.85*
**Mean**	**0.07**	**21.11**	**65.18**	**2011.96**	**63,251.84**	**4.12**	**13,117.87**	**1806.17**	**0.27**
LC-FA	15.31	138.51	147.16	23,378.88	57,161.72	35.05	19,327.39	1682.51	7.66
LC-FA	15.41	129.40	141.47	23,528.77	57,804.23	34.23	19,329.03	1670.63	7.71
LC-FA	15.18	147.23	141.17	23,208.17	56,357.05	34.32	19,074.44	1636.03	7.31
LC-FA	17.87	146.15	139.22	24,662.80	48,740.05	34.75	17,776.86	1540.45	7.10
LC-FA	17.12	135.49	138.98	24,069.92	48,977.57	34.50	17,825.53	1548.15	6.96
LC-FA	17.31	137.42	142.60	24,450.79	49,207.40	34.91	17,926.68	1536.00	6.41
**Mean**	**16.37**	**139.03**	**141.77**	**23,883.22**	**53,041.34**	**34.63**	**18,543.32**	**1602.30**	**7.19**
LC—CA	11.01	68.46	57.78	20,387.61	29,882.33	26.47	13,505.60	814.11	5.41
LC—CA	11.09	64.93	67.65	21,140.42	29,373.36	26.25	13,479.66	827.43	5.02
LC—CA	11.31	65.62	90.08	20,935.40	29,795.32	26.57	13,782.47	830.92	2.64
LC—CA	11.00	66.97	discarded	20,091.15	28,836.86	26.55	13,652.25	842.80	2.11
LC—CA	11.49	62.38	85.98	21,120.47	29,358.79	26.37	14,187.62	873.27	1.74
LC—CA	11.23	63.97	84.86	20,485.97	29,649.48	26.86	14,126.96	839.43	1.83
**Mean**	**11.19**	**65.39**	**77.27**	**20,693.50**	**29,482.69**	**26.51**	**13,789.09**	**837.99**	**3.12**

**Table 7:** Raw data and mean values for TE measured by ICP-OES. Values marked with * were at detection limit. All values relate to mg/kg DM.

**Material**	**Na**	**Ni**	**Pb**	**Sb**	**Se**	**Sr**	**Ti**	**Tl**	**V**
OFMSW	5450.51	4.74	71.58	0.02*	0.02*	59.97	269.59	3.43	10.41
OFMSW	5290.73	5.22	31.62	0.02*	0.02*	70.66	240.18	0.13	12.36
OFMSW	5292.78	6.03	40.05	0.02*	0.02*	53.92	224.84	0.02*	10.21
OFMSW	5231.04	6.01	30.13	0.02*	0.02*	47.84	228.86	0.02*	9.66
**Mean**	**5316.27**	**5.50**	**43.34**	**0.02**	**0.02**	**58.10**	**240.87**	**0.90**	**10.66**
SEWS	6905.54	20.15	31.76	4.26	0.02*	388.72	296.26	0.02*	54.55
SEWS	7396.05	20.91	34.55	4.27	0.02*	403.48	275.34	0.02*	56.38
SEWS	8069.46	22.20	30.78	4.34	0.02*	383.98	299.81	0.02*	53.69
SEWS	7476.69	22.06	27.80	4.12	0.02*	377.65	275.62	0.02*	52.10
**Mean**	**7461.93**	**21.33**	**31.22**	**4.25**	**0.02**	**388.46**	**286.76**	**0.02**	**54.18**
HC-FA	1660.36	315.15	9.35	0.06*	1.07	557.87	55.98	3.08	0.65
HC-FA	1525.70	113.65	9.98	0.06*	0.73	548.78	59.00	1.59	0.37
HC-FA	1704.63	225.55	9.01	0.06*	0.45	536.36	49.55	1.48	0.71
HC-FA	1292.71	91.31	6.49	0.05*	0.05*	417.53	49.20	1.45	1.55
**Mean**	**1545.85**	**186.41**	**8.71**	**0.06**	**0.57**	**515.13**	**53.43**	**1.90**	**0.82**
HC-RA	1424.04	40.97	1.34	0.04*	0.04*	285.21	36.71	0.27	1.14
HC-RA	4093.05	147.77	1.61	0.12*	0.12*	750.50	92.37	1.74	0.31
HC-RA	1750.00	70.09	0.98	0.06*	0.06*	358.14	42.70	1.10	1.65
HC-RA	1800.32	69.07	1.69	0.05*	0.42	349.36	13.80	1.43	3.84
**Mean**	**2266.85**	**81.98**	**1.41**	**0.07**	**0.16**	**435.80**	**46.39**	**1.13**	**1.73**
LC-FA	3674.92	35.18	417.82	16.47	0.03*	429.70	2523.10	0.03*	55.41
LC-FA	3721.56	34.42	426.59	16.40	0.03*	423.28	2558.86	0.03*	55.32
LC-FA	3609.35	33.00	400.86	15.91	0.03*	404.81	2511.20	0.03*	55.40
LC-FA	4167.77	36.51	426.72	18.60	0.03*	364.06	3227.12	0.03*	57.86
LC-FA	4058.38	44.95	464.05	20.65	0.03*	376.32	3031.00	0.03*	55.44
LC-FA	4153.90	35.01	420.41	19.02	0.03*	371.53	3280.71	0.03*	57.23
**Mean**	**3897.65**	**36.51**	**426.07**	**17.84**	**0.03**	**394.95**	**2855.33**	**0.03**	**56.11**
LC—CA	2846.41	38.07	35.33	1.12	0.03*	247.17	2302.57	0.03*	41.89
LC—CA	2788.39	40.16	33.43	0.66	0.03*	254.59	2355.97	0.03*	42.09
LC—CA	2816.41	28.71	27.29	0.76	0.03*	259.89	2451.88	0.03*	43.34
LC—CA	2905.88	28.17	discarded	3.43	0.03*	295.64	2288.97	0.03*	42.47
LC—CA	2929.23	23.77	42.23	0.86	0.03*	302.86	2354.84	0.03*	43.98
LC—CA	2888.05	27.64	58.58	2.06	0.03*	303.95	2393.28	0.03*	43.28
**Mean**	**2862.40**	**31.09**	**39.37**	**1.48**	**0.03**	**277.35**	**2357.92**	**0.03**	**42.84**

**Table 8:** Raw data and mean values for TE measured by ICP-OES. Values marked with * were at detection limit. All values relate to mg/kg DM.

**Material**	**Zn**	**Ga**	**In**	**Si**	**P**	**S**
OFMSW	96.07	12.40	13.31	5870.12	3350.59	2237.54
OFMSW	88.10	11.72	13.03	7395.75	7129.32	2006.61
OFMSW	98.57	10.34	10.50	5553.36	3145.71	2157.90
OFMSW	85.74	8.74	5.60*	6007.33	2792.38	2040.56
**Mean**	**92.12**	**10.80**	**10.61**	**6206.64**	**4104.50**	**2110.65**
SEWS	1297.14	29.46	5.98*	6353.54	35,411.09	12,630.98
SEWS	1338.11	29.29	6.13*	5922.67	37,039.94	13,091.43
SEWS	1273.69	30.51	6.18*	6265.19	36,345.70	12,613.85
SEWS	1247.60	30.67	6.03*	6855.68	35,414.26	12,676.11
**Mean**	**1289.13**	**29.98**	**6.08**	**6349.27**	**36,052.75**	**12,753.09**
HC-FA	1241.42	15.92*	124.67	2052.66	4837.87	866.12
HC-FA	623.01	16.46*	118.18	1917.38	4523.87	740.36
HC-FA	953.67	17.44	115.77	1945.30	4348.78	842.82
HC-FA	566.93	13.44*	101.85	1414.09	3419.58	592.78
**Mean**	**846.26**	**15.81**	**115.12**	**1832.36**	**4282.52**	**760.52**
HC-RA	154.29	12.02*	46.29	1571.49	2866.85	643.16
HC-RA	486.48	33.37*	125.68	4150.12	7745.66	1816.87
HC-RA	157.97	15.53*	60.22	2028.29	3827.37	872.06
HC-RA	230.93	14.25*	8.05*	discarded	4308.79	1182.94
**Mean**	**257.42**	**18.79**	**60.06**	**1941.03**	**4687.17**	**1128.76**
LC-FA	1908.58	n.a.	n.a.	n.a.	n.a.	n.a.
LC-FA	1927.25	n.a.	n.a.	n.a.	n.a.	n.a.
LC-FA	1781.62	n.a.	n.a.	n.a.	n.a.	n.a.
LC-FA	1910.48	n.a.	n.a.	n.a.	n.a.	n.a.
LC-FA	2156.99	n.a.	n.a.	n.a.	n.a.	n.a.
LC-FA	1968.30	n.a.	n.a.	n.a.	n.a.	n.a.
**Mean**	**1942.20**	**n.a.**	**n.a.**	**n.a.**	**n.a.**	**n.a.**
LC—CA	291.53	n.a.	n.a.	n.a.	n.a.	n.a.
LC—CA	305.28	n.a.	n.a.	n.a.	n.a.	n.a.
LC—CA	270.44	n.a.	n.a.	n.a.	n.a.	n.a.
LC—CA	450.46	n.a.	n.a.	n.a.	n.a.	n.a.
LC—CA	343.65	n.a.	n.a.	n.a.	n.a.	n.a.
LC—CA	317.20	n.a.	n.a.	n.a.	n.a.	n.a.
**Mean**	**329.76**	**n.a.**	**n.a.**	**n.a.**	**n.a.**	**n.a.**

### 2.5 Digestion experiments

The volumetric biogas production was measured using glass manometers whenever the manometer functioning as 1 L gas storage was nearly full, considering the temperature within the digester (2-L insulated glass vessel) and ambient conditions. After several days, the CH_4_ concentration was analyzed with a portable biogas analyzer. Specific biogas and CH_4_ productions were related to oDM and calculated for standard conditions (1013 hPa, 0 °C, dry gas). Experiments were conducted in two batch test series for 40 days (series 1) and 42 days (series 2) in triplicate and duplicate, respectively (set-ups visible in Table 9 and Table 12). All tests were carried out according to [5].

**Table 9:** Experimental set-up in test series 1 (operating temperature 35 °C, reactors stirred 60 s/h). oDM delivered by LC—CA and LC-FA (Table 3) was neglected.

**Digester**	**Variant**	**Inoculum**	**Additional feedstock** **[g DM]**	**Retention time** **[d]**	**Total DM_Digester_** **[g]**	**Total oDM_Digester_** **[g]**
1	SEWS (blank)	2 L SEWS	–	40	79.56 + 0	50.99 + 0
2	SEWS (blank)	2 L SEWS	–	40	79.56 + 0	50.99 + 0
3	SEWS (blank)	2 L SEWS	–	40	79.56 + 0	50.99 + 0
4	SEWS + LC—CA	2 L SEWS	16.72 (LC—CA)	40	79.56 + 16.72	50.99 + 0
5	SEWS + LC—CA	2 L SEWS	15.12 (LC—CA)	40	79.56 + 15.12	50.99 + 0
6	SEWS + LC—CA	2 L SEWS	16.43 (LC—CA)	40	79.56 + 16.43	50.99 + 0
7	SEWS + LC-FA	2 L SEWS	14.04 (LC-FA)	40	79.56 + 14.04	50.99 + 0
8	SEWS + LC-FA	2 L SEWS	13.93 (LC-FA)	40	79.56 + 13.93	50.99 + 0
9	SEWS + LC-FA	2 L SEWS	14.59 (LC-FA)	40	79.56 + 14.59	50.99 + 0

**Table 10:** Measured CH_4_ concentrations [%] and weighted mean values for each digester (D1-D9) in test series 1.

**Time** **[d]**	**D-1**	**D-2**	**D-3**	**D-4**	**D-5**	**D-6**	**D-7**	**D-8**	**D-9**
	**CH_4_**	**CH_4_**	**CH_4_**	**CH_4_**	**CH_4_**	**CH_4_**	**CH_4_**	**CH_4_**	**CH_4_**
**0**	0	0	0	0	0	0	0	0	0
**6.10**	64.83	65.37	64.76	65.85	65.62	65.06	71.92	71.87	72.08
**39.86**	67.94	68.00	67.96	68.09	68.27	69.65	70.61	72.26	73.29
**Weighted mean**	**66.04**	**66.37**	**65.89**	**66.66**	**66.63**	**66.54**	**71.42**	**72.02**	**72.60**

In series 1, the influence of LC coarse and LC fly ashes on the anaerobic digestion process was determined (Table 11). In series 2, multiple configurations of OFMSW as a baseline feedstock mixed with LC and HC ashes at different ratios were tested (Table 14 and Table 15). Based on the data of series 1, LC coarse ash was used instead of LC fly ash. The remaining oDM content of HC ashes was neglected in calculations as it is considered to be not available for microorganisms. For both series, blind variants were carried out, determining the residual biogas potential of the digested sewage sludge/inoculum.

**Table 11:** Biogas (Bg) and CH4 yields (cumulative value) for each time-step and digester (D1-D9) in test series 1. All values related to mL/g oDM for standard conditions (1013 hPa, 0 °C, dry gas).

**Time** **[d]**	**D-1**		**D-2**		**D-3**		**D-4**		**D-5**		**D-6**		**D-7**		**D-8**		**D-9**	
	**Bg**	**CH_4_**	**Bg**	**CH_4_**	**Bg**	**CH_4_**	**Bg**	**CH_4_**	**Bg**	**CH_4_**	**Bg**	**CH_4_**	**Bg**	**CH_4_**	**Bg**	**CH_4_**	**Bg**	**CH_4_**
**0**	0	0	0	0	0	0	0	0	0	0	0	0	0	0	0	0	0	0
**0.11**	3	2	4	2	3	2	4	3	4	3	5	3	3	2	3	2	3	2
**0.22**	5	3	5	3	5	3	7	4	7	5	7	5	4	3	4	3	4	3
**0.74**	21	14	21	14	23	15	24	16	26	17	26	17	16	12	19	13	16	12
**1.03**	27	17	27	18	31	20	31	20	33	22	33	21	23	17	25	18	22	16
**1.29**	32	20	34	22	36	23	37	25	39	25	41	27	27	19	30	21	25	18
**1.77**	43	28	46	30	45	29	48	32	48	32	53	34	34	24	37	27	33	24
**2.11**	50	33	53	34	53	34	55	36	54	35	61	39	41	29	43	31	39	28
**2.80**	61	40	66	43	63	41	67	44	64	42	73	48	46	33	51	37	46	33
**3.00**	65	42	72	47	69	45	71	47	68	45	79	51	51	36	54	39	49	35
**3.93**	74	48	84	55	79	51	82	54	77	50	89	58	55	40	60	43	55	40
**4.97**	82	53	96	63	88	57	92	61	86	56	99	64	59	43	66	48	61	44
**6.10**	90	59	105	68	96	62	100	66	93	61	106	69	63	45	72	52	67	48
**7.01**	94	61	110	72	101	65	105	69	98	64	112	73	65	47	75	54	70	50
**7.88**	98	64	115	75	104	68	109	72	102	67	115	75	69	49	77	56	73	53
**8.84**	101	66	119	78	109	71	113	74	106	70	119	78	70	51	80	58	77	56
**10.23**	106	69	124	82	113	74	118	78	110	73	125	82	74	53	85	61	81	59
**11.19**	109	71	127	84	116	76	121	80	113	75	127	84	76	54	88	63	83	60
**12.08**	111	73	130	85	119	78	123	82	116	77	129	85	77	55	89	64	85	62
**13.07**	115	75	133	88	122	80	125	83	119	79	132	87	80	57	91	65	87	63
**14.08**	117	77	135	89	124	81	128	85	121	80	134	89	82	58	93	67	90	65
**15.86**	120	79	140	93	128	84	131	87	125	83	138	91	85	61	96	69	92	67
**17.95**	125	82	144	95	131	86	135	89	128	85	141	93	89	63	99	71	96	70
**20.94**	128	84	150	100	135	89	139	92	133	88	145	96	92	66	103	74	100	72
**26.96**	136	90	158	105	141	93	146	97	140	93	150	99	97	69	109	79	106	77
**34.98**	144	95	166	110	145	95	152	102	148	98	154	102	101	72	116	83	113	82
**39.86**	**148**	**98**	**169**	**112**	**149**	**98**	**156**	**104**	**150**	**100**	**156**	**104**	**102**	**73**	**119**	**86**	**116**	**84**

**Table 12:** Experimental set-up in test series 2 (operating temperature 35 °C, reactors stirred 60 s/h). oDM contents delivered by ashes (Table 3) were neglected as they were either negligible (LC) or “not available for microorganisms” (HC).

**Digester**	**Variant**	**Inoculum**	**Additional feedstock** **[g DM]**	**Retention time** **[d]**	**Total DM_Digester_** **[g]**	**Total oDM_Digester_** **[g]**
1	SEWS (blank)	1 L SEWS + 1 L tap water	–	42	32.60 + 0	20.56 + 0
2	SEWS (blank)	1 L SEWS + 1 L tap water	–	42	32.60 + 0	20.56 + 0
3	SEWS + OFMSW	1 L SEWS + 1 L tap water	9.82 (OFMSW)	42	32.60 + 9.82	20.56 + 7.66
4	SEWS + OFMSW	1 L SEWS + 1 L tap water	9.50 (OFMSW)	42	32.60 + 9.50	20.56 + 7.41
5	SEWS + OFMSW + LC—CA (1:1)	1 L SEWS + 1 L tap water	9.59 (OFMSW) 9.87 (LC—CA)	42	32.60 + 9.59 + 9.87	20,56 + 7.48 + 0
6	SEWS + OFMSW + LC—CA (1:1)	1 L SEWS + 1 L tap water	8.96 (OFMSW) 9.94 (LC—CA)	42	32.60 + 8.96 + 9.94	20.56 + 6.99 + 0
7	SEWS + OFMSW + LC—CA (1:3)	1 L SEWS + 1 L tap water	9.54 (OFMSW) 32.13 (LC—CA)	42	32.60 + 9.54 + 32.13	20.56 + 7.44 + 0
8	SEWS + OFMSW + LC—CA (1:3)	1 L SEWS + 1 L tap water	9.86 (OFMSW) 32.57 (LC—CA)	42	32.60 + 9.86 + 32.57	20.56 + 7.69 + 0
9	SEWS + OFMSW + LC—CA (1:10)	1 L SEWS + 1 L tap water	9.70 (OFMSW) 92.05 (LC—CA)	42	32.60 + 9.70 + 92.05	20.56 + 7.56 + 0
10	SEWS + OFMSW + LC—CA (1:10)	1 L SEWS + 1 L tap water	8.69 (OFMSW) 90.19 (LC—CA)	42	32.60 + 8.69 + 90.19	20.56 + 6.78 + 0
11	SEWS + OFMSW + HC-FA (1:1)	1 L SEWS + 1 L tap water	8.77 (OFMSW) 9.11 (HC-FA)	42	32.60 + 8.77 + 9.11	20.56 + 6.84 + 0
12	SEWS + OFMSW + HC-FA (1:1)	1 L SEWS + 1 L tap water	9.04 (OFMSW) 8.97 (HC-FA)	42	32.60 + 9.04 + 8.97	20.56 + 7.05 + 0

**Table 13:** Measured CH4 concentrations [%] and weighted mean values for each digester (D1-D12) in test series 2.

**Time** **[d]**	**D-1**	**D-2**	**D-3**	**D-4**	**D-5**	**D-6**	**D-7**	**D-8**	**D-9**	**D-10**	**D-11**	**D-12**
	**CH_4_**	**CH_4_**	**CH_4_**	**CH_4_**	**CH_4_**	**CH_4_**	**CH_4_**	**CH_4_**	**CH_4_**	**CH_4_**	**CH_4_**	**CH_4_**
**0**	0	0	0	0	0	0	0	0	0			
**26.97**	71.94	72.66	64.57	64.74	68.61	68.61	75.80	78.98	96.57	–	71.22	71.66
**41.95**	74.19	74.88	71.55	68.23	72.82	72.82	75.09	76.19	97.02	98.51	69.26	71.01
**Weighted mean**	72.25	72.98	65.08	65.03	68.98	68.98	75.75	78.72	96.71	98.51	70.96	71.57

**Table 14:** Biogas (Bg) and CH_4_ yields (cumulative value) for each time-step and digester (D1-D8) in test series 2. All values related to mL/g oDM for standard conditions (1013 hPa, 0 °C, dry gas).

**Time** **[d]**	**D-1**		**D-2**		**D-3**		**D-4**		**D-5**		**D-6**		**D-7**		**D-8**	
	**Bg**	**CH_4_**	**Bg**	**CH_4_**	**Bg**	**CH_4_**	**Bg**	**CH_4_**	**Bg**	**CH_4_**	**Bg**	**CH_4_**	**Bg**	**CH_4_**	**Bg**	**CH_4_**
**0**	0	0	0	0	0	0	0	0	0	0	0	0	0	0	0	0
**0.33**	5	3	5	4	7	4	8	5	7	5	6	4	5	4	3	3
**0.90**	14	10	14	10	28	18	28	18	28	19	25	17	20	15	14	11
**1.38**	19	13	19	14	44	28	44	28	44	30	40	27	31	23	23	18
**1.96**	23	16	24	17	60	39	62	40	62	42	55	38	44	33	36	28
**2.27**	25	18	25	18	67	43	71	46	69	47	62	42	49	37	42	33
**2.98**	30	21	31	22	79	51	84	55	82	56	74	51	60	45	55	43
**4.10**	36	26	37	27	94	61	100	65	99	68	89	61	73	56	69	55
**5.98**	46	33	46	33	113	73	118	77	119	82	108	74	92	70	87	69
**7.34**	52	37	52	38	124	80	129	83	130	89	118	81	103	78	98	78
**8.01**	54	39	54	39	129	83	134	87	135	92	122	84	108	82	103	82
**9.01**	58	42	58	42	135	87	139	90	141	96	128	88	114	86	109	86
**10.01**	62	44	61	45	140	91	145	94	146	100	133	92	120	91	115	91
**14.05**	73	52	72	52	153	99	159	103	162	111	148	101	135	102	129	102
**16.24**	77	56	77	56	159	103	165	107	168	116	154	106	141	107	135	106
**19.90**	85	61	84	61	166	107	173	112	176	121	161	110	148	112	142	112
**23.96**	92	66	92	67	173	111	181	117	184	126	168	115	154	117	148	117
**26.97**	97	70	96	70	176	114	185	120	189	130	171	117	159	120	152	120
**30.26**	101	73	100	73	180	116	190	123	193	133	175	120	162	123	156	123
**34.08**	105	76	105	76	183	119	194	126	197	136	178	122	166	126	160	126
**37.25**	108	78	108	79	186	121	197	128	201	138	181	124	168	127	163	128
**41.95**	**113**	**81**	**113**	**82**	**190**	**124**	**202**	**131**	**207**	**143**	**184**	**127**	**172**	**131**	**168**	**132**

**Table 15:** Biogas (Bg) and CH_4_ yields (cumulative value) for each time-step and digester (D9-D12) in test series 2. All values related to mL/g oDM for standard conditions (1013 hPa, 0 °C, dry gas).

**Time** **[d]**	**D-9**		**D-10**		**D-11**		**D-12**	
	**Bg**	**CH_4_**	**Bg**	**CH_4_**	**Bg**	**CH_4_**	**Bg**	**CH_4_**
**0**	0	0	0	0	0	0	0	0
**0.33**	0	0	0	0	5	4	5	4
**0.90**	0	0	0	0	19	14	18	13
**1.38**	0	0	0	0	30	21	30	21
**1.96**	0	0	2	2	43	31	43	31
**2.27**	0	0	2	2	49	35	48	35
**2.98**	1	1	4	4	59	42	59	42
**4.10**	2	2	6	6	72	51	71	51
**5.98**	3	3	9	9	89	63	88	63
**7.34**	5	4	11	11	98	70	98	70
**8.01**	5	5	12	12	102	73	102	73
**9.01**	6	6	13	13	108	77	108	77
**10.01**	8	7	14	14	113	80	113	81
**14.05**	11	11	16	16	130	93	129	93
**16.24**	15	14	17	17	137	98	137	98
**19.90**	35	34	18	17	146	104	146	105
**23.96**	61	59	20	19	154	110	154	111
**26.97**	70	67	23	23	160	114	160	115
**30.26**	77	75	31	31	166	118	166	119
**34.08**	90	87	54	53	172	122	173	124
**37.25**	96	92	65	64	177	126	178	127
**41.95**	**102**	**98**	**71**	**70**	**184**	**130**	**185**	**133**

## Declaration of Competing Interest

The authors declare that they have no known competing financial interests or personal relationships, which have, or could be perceived to have, influenced the work reported in this article.
